# The restricted adhesion of bone marrow mesenchymal stem cells by stepped structures on surfaces of hydroxyapatite†

**DOI:** 10.1039/d2ra00756h

**Published:** 2022-04-20

**Authors:** Jin Chen, Zhuo Huang, Fang Wang, Min Gong, Xueli Zhang, Yajing Wang, Zuquan Hu, Zhu Zeng, Yun Wang

**Affiliations:** Key Laboratory of Biology and Medical Engineering/Immune Cells and Antibody Engineering Research Center of Guizhou Province, School of Biology and Engineering, Guizhou Medical University Guiyang 550025 P. R. China huzuquan@gmc.edu.cn zengzhu@gmc.edu.cn wangyun@gmc.edu.cn; Key Laboratory of Infectious Immune and Antibody Engineering of Guizhou Province, School of Basic Medical Sciences, Guizhou Medical University Guiyang 550025 P. R. China; The Affiliated Stomatological Hospital of Guizhou Medical University, Guizhou Medical University Guiyang 550025 P. R. China

## Abstract

Currently, many researches have developed several strategies to design the surface structures of hydroxyapatite (HA), and have proved that the surface structures are pivotal in guiding the adhesion of bone marrow mesenchymal stem cells (BMSCs) as well as subsequent cellular behaviours. Most of these strategies, such as altering roughness and constructing surface patterning of HA, involve the construction of geometric topographies at the micro/nanoscale. However, besides geometric topographies, crystal defects are also important characteristics of surface structures and would alter many local physicochemical properties, which is critical for contact between cells and bioceramic surfaces. For the practical applications of crystal defects, a major hindrance is that crystal defects are usually unstable and easily eliminated during crystallization, which limits the large-scale fabrication of materials with crystal defects. In this work, given that stepped structures contain massive stable crystal defects on their step edges and kinks, we proposed a feasible and efficient method to fabricate HA dishes with stepped structures on their surfaces. First, plate-like HA mesocrystals were prepared from CaHPO_4_*via* topotactic transformation, and were shaped into HA dishes by vacuum-filtration. Then, a sintering process was applied to facilitate the formation of stepped structures on the surfaces. We demonstrated that the generation of stepped structures could restrict the adhesion of BMSCs and showed the restriction effect is highly correlated with the density of exposed stepped structures. This phenomenon is interesting and the construction of a cell adhesion model is robust and easy, the underlying mechanisms of which deserve further exploration. Furthermore, constructing stepped structures on surfaces may be a new useful strategy to regulate cell adhesion and could also cooperate with other methods that do not need change in the surface crystal structure.

## Abstract 2

Stepped structures largely exposed on surfaces of HA significantly restrict the adhesion of bone marrow mesenchymal stem cells.

## Graphical abstract

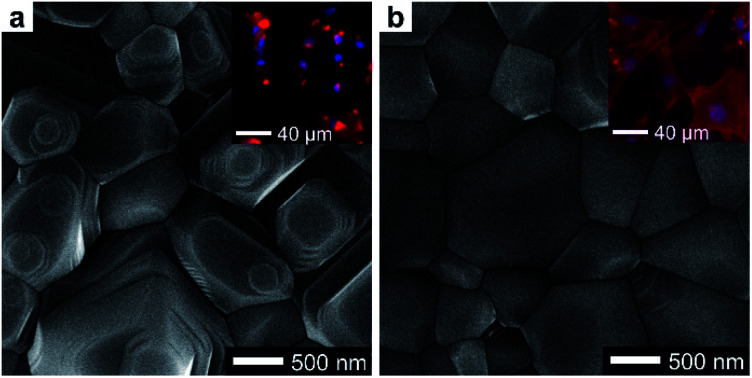

## Introduction

Hydroxyapatite (HA), as a classical bioceramic material, is widely used in bone repair and interface modification of implant materials.^1^ After implantation, the performance of HA materials is largely determined by their interaction outcomes with bone marrow mesenchymal stem cells (BMSCs) that possess multi-lineage differentiation ability and can secrete anti-inflammatory cytokines to influence the functions of immune cells.^2,3^ During the response process of BMSCs to biomaterials, cell adhesion is the first occurring cellular behaviour and could be classified into three categories: focal complexes, focal adhesions and fibrillar adhesions.^4^ Focal complexes are early adhesions and could transform into focal adhesions.^5^ Following actomyosin contraction, focal adhesions finally develop into fibrillar adhesions.^6^ During this process, many mechanochemical signal transductions could be activated or inhibited, leading to huge influences on subsequent cellular behaviours, such as migration, proliferation, differentiation and self-renewal.^1,7,8^ Recently, numerous reports have proved that designing surface structures of materials is an effective way to guide cell fate, which has become an extremely hot and competitive research area.^9,10^ Many strategies have been proposed to construct geometric topographies on HA surfaces in micro/nanoscale, such as altering roughness by polishing or etching, and constructing surface patterning *via* photolithography or self-assemble technology.^11–14^ All these strategies are successful and realize the modulation of cell adhesion as well as subsequent cellular behaviours. However, for HA and other crystallized bioceramics, their surface structures are characterized not only by geometric topographies but also by surface crystal structures.^15–20^ Some theoretical calculations and studies have shown that crystal defects on HA surfaces are high reactive and can affect many local physicochemical properties due to the locally varying stoichiometric ratio and the special atomic coordination of defect sites, a typical example of which is the capacity of material surfaces to adsorb proteins.^1,15,16,18,21,22^ It is well known that cell adhesion is usually mediated by the binding of cytomembrane receptors (*e.g.*, integrins) to the adsorbed proteins on the material surface;^8,9,23^ this indicates that creating crystal defects on HA surfaces may be a new way to modulate the adhesion of BMSCs.

Surface crystal defects, especially the isolated defects, are unstable and easily eliminated during the interface relaxation or further recrystallization because of the minimization of surface energy.^24,25^ Therefore, obtaining a well-designed HA surface structure with a large number of stable crystal defects is the major issue for the study on their effects on cell adhesion. Stepped structures, also known as terrace-step structures, are a kind of planar defects and formed on grain surfaces during crystal growth, which contain a large number of crystal defects on their step edge and kink sites.^26–28^ These crystal defects are relatively stable due to the Ehrlich–Schwoebel barrier between each terrace.^28–30^ Thus, it will be a very interesting and promising attempt to construct stepped structures on HA surfaces and figure out their effects on cell adhesion. However, most conventional methods designed for the construction of stepped structures are based on classical crystal growth pathways, such as screw dislocation-driven growth, 2D nucleation, and epitaxial growth.^31–33^ For these methods, accurate control on low supersaturation is essentially required for the atom-by-atom crystal growth process and therefore, some complex equipment and harsh reaction conditions are usually needed. Few of the methods can afford facile and massive preparation of HA materials with stepped structures on surfaces.

Oriented attachment, usually referred as a nonclassical or a particle-by-particle crystal growth pathway, has been proven to be a promising way for production of crystallized materials with planar defects.^34–36^ In previous work, inspired by the principle of oriented attachment, we proposed a feasible method to create stepped structures on surfaces of HA particles.^37,38^ However, it just provided a way to prepare micron-scale HA particles with stepped structures on surfaces. To utilize HA for cell adhesion and follow-up research, the method to fabricate macro-scale HA materials with stepped structures on surfaces is very necessary, but has not yet been developed. In the current work, we developed and adapted the previous method to prepare HA dishes with stepped structures on surfaces. This method is robust, easy and suitable for large-scale production. We demonstrated that the stepped structures could restrict the adhesion of BMSCs and showed the restriction effect is highly correlated with the density of stepped structures. Our strategy tried to affect cell adhesion by only altering surface structures of HA. Theoretically, this strategy should be able to cooperate with other developed strategies to further effectively and accurately regulate cell adhesion as well as subsequent cell behaviours.

## Results and discussion

**Fig. 1:**
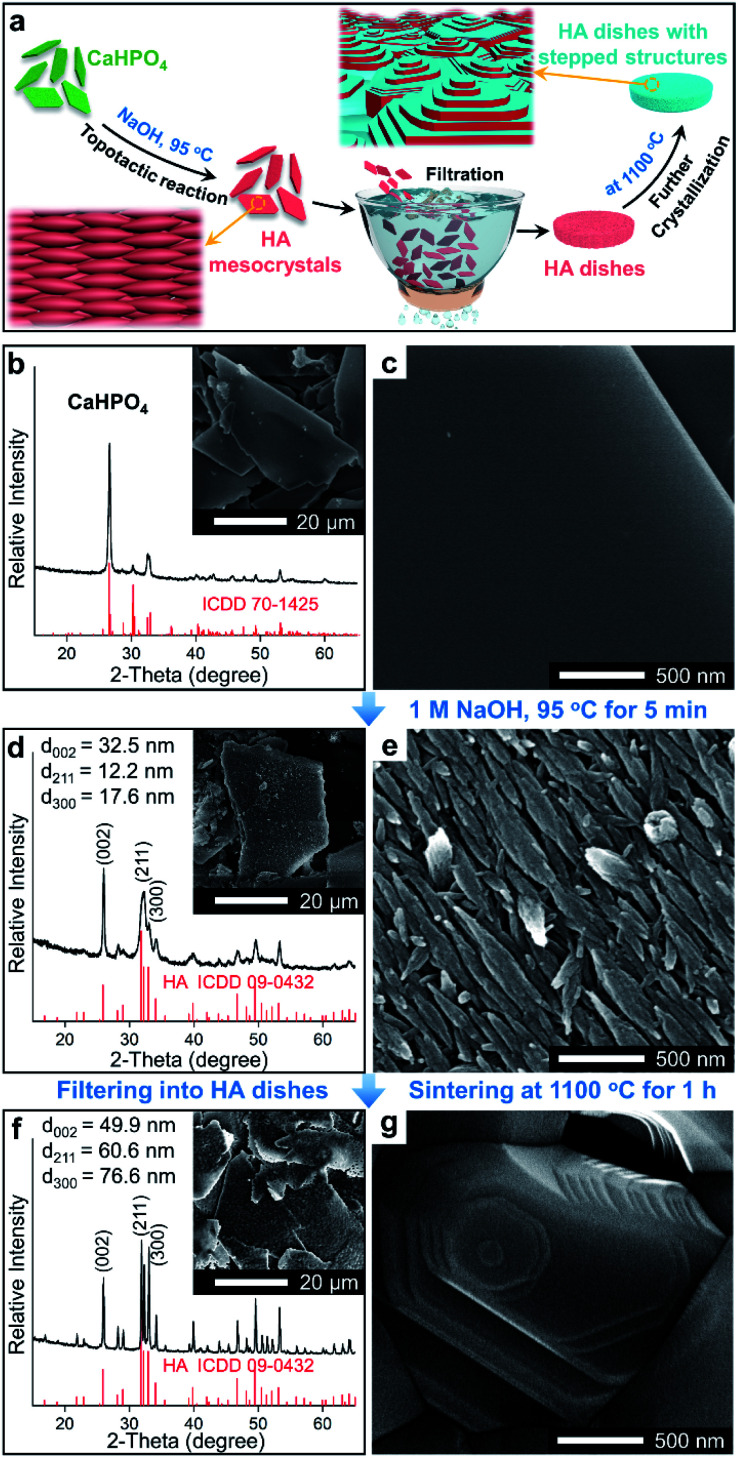
(a) Schematic diagram of the formation process of the HA dishes with stepped structures on surfaces. (b)–(g) SEM images and XRD patterns of samples: (b) and (c) CaHPO_4_ particles; (d) and (e) HA mesocrystals; (f) and (g) HA dishes with the stepped structures on surfaces.

In this work, a method based on oriented attachment was applied to fabricate HA dishes with stepped structures on exposed surfaces (Fig. 1a). In brief, the HA mesocrystals were first prepared *via* a topotactic transformation reaction from CaHPO_4_ to HA. Then, the obtained HA mesocrystals were dispersed in distilled water and vacuum-filtered to prepare HA dishes. Finally, a sintering process at 1100 °C was used to facilitate the crystallization, coarsening and fusion of the nanoscale building units of the HA mesocrystals. During this process, stepped structures were formed on the exposed surfaces of HA dishes because of the accumulation and evolution of minor crystalline mismatches between the nanoscale building units.^37^

**Fig. 2:**
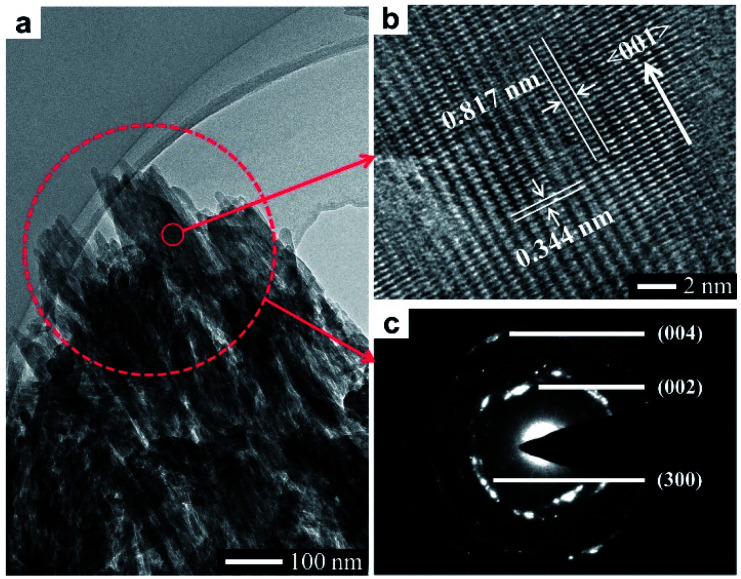
TEM analysis of a typical HA mesocrystal: (a) TEM image; (b) HRTEM image; (c) SAED pattern.

The homemade CaHPO_4_ particles are elongated plates with a length of ∼40 μm (Fig. 1b) and a thickness of ∼700 nm (Fig. S1†), their surfaces are very smooth and without any signs of small grains (Fig. 1c). To obtain HA mesocrystals, the CaHPO_4_ particles were added into 1 M NaOH solution (pre-heated to 95 °C) for 5 min. The XRD pattern (Fig. 1d) matches the standard pattern of HA (ICDD 09-0432), conforming the transformation of CaHPO_4_ to HA after the topotactic transformation. The low-magnification SEM image (Fig. 1d) shows that the overall shape of HA particles remains the same after transformation, while their surfaces become rough. High magnification SEM image (Fig. 1e) indicates that the rough surfaces comprise largely well-arranged spindle-like nanoparticles with a length of 200–500 nm. Furthermore, there are some dislocations and gaps between these nanoparticles, which is caused by the shrinkage during the topotactic transformation. The TEM images (Fig. 2a and b) confirm that the spindle-like nanoparticles mainly parallel to each other. The SAED pattern (Fig. 2c) shows a “single-crystal-like” diffraction spots with slight arcs, which is typical for mesocrystals. The TEM analysis suggests that the spindle-like nanoparticles are crystallographically aligned along the [001] direction of HA crystal and with some small misalignments.^39^ All above results indicate that the resulting micron-scale HA plates are mesocrystal particles, which are built of crystallographically aligned spindle-like particles with some small misalignments. According to the previous work,^37^ these mismatches existing in HA mesocrystals are essential for the following formation of stepped structures.

**Fig. 3:**
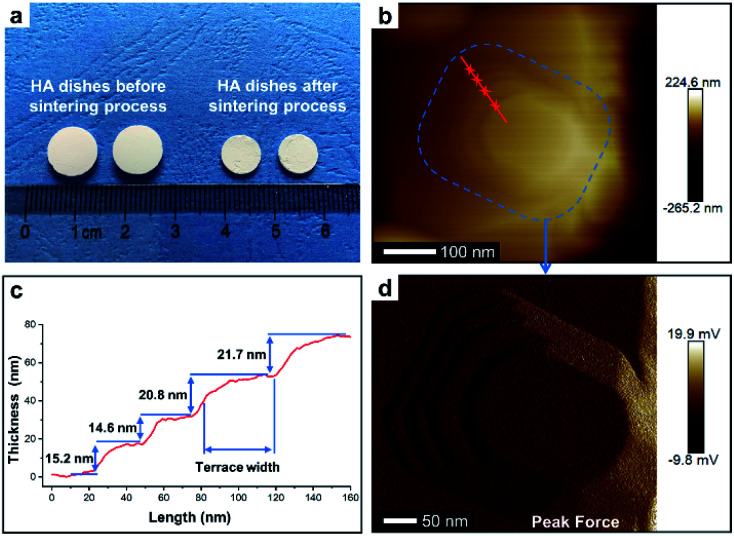
(a) Digital photo of HA dishes before and after the sintering process (1100 °C for 1 h). (b) AFM image of a typical stepped structure on surface of HA dish. (c) AFM height profile along the dashed red line in (b). (d) AFM peak force mode image of blue area in (b).

To perform subsequent cell adhesion experiments, macro-scale HA materials with stepped structures on surfaces need to be obtained. Thus, the micron-scale and plate-like HA mesocrystals, as the raw materials, were stacked into parallel layers *via* a vacuum-filtrating process to prepare HA dishes. Then, a follow-up sintering process (1100 °C for 1 h) was used to promote the formation of stepped structures. As shown in Fig. 3a, the HA dishes shrink obviously after the sintering process, indicating that the HA dishes become denser and their crystallinities increase. The XRD pattern (Fig. 1f) indicates that the sintering process does not change the chemical composition or introduce any impurity phase. The calculation results (Fig. 1f) according to Scherrer equation indicate that the crystallite sizes along all directions are increased significantly, confirming the increases in the crystallinities of HA dishes. The SEM images indicate that the spindle-like nanoparticles in the HA mesocrystals (Fig. 1e) disappear and grow into much larger grains with visible stepped structures exposed on surfaces (Fig. 1g). The AFM measurements (Fig. 3b–d) clearly display each terrace plane of the stepped structures. The terrace width is ∼40 nm and their interlayer step height ranges from 15.2 to 21.7 nm. All above results indicate that pure HA dishes with largely stepped structures on surfaces were successfully prepared *via* a vacuum-filtration and a follow-up sintering process.

**Fig. 4:**
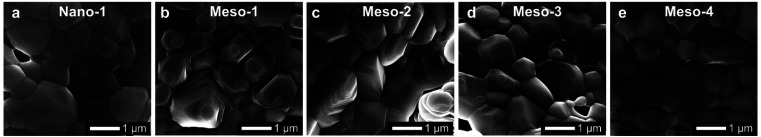
SEM images of different HA dish samples: (a) Nano-1, (b) Meso-1, (c) Meso-2, (d) Meso-3 and (e) Meso-4.

To investigate whether the exposure of the stepped structures would affect the adhesion of cells, we prepared HA dishes without stepped structures (*i.e.*, the surfaces of grains are smooth), which was set as the control group. Briefly, HA nanoparticles with a length of 100–400 nm (Fig. S2a†) were synthesized, which are similar to the spindle-like nanoparticles of HA mesocrystals (Fig. 1e). Then, HA dishes built of randomly arranged nanoparticles were prepared through a vacuum-filtrating process, and a follow-up sintering process (1100 °C for 1 h) was applied. The HA dishes are labelled as Nano-1. The XRD patterns (Fig. S2b†) show that the Nano-1 sample is pure HA after the sintering process, and its crystallization degree is close to that of Meso-1 sample. The low-magnification (Fig. S2c†) and high-magnification (Fig. 4a and S2d†) SEM images show that the surfaces of Nano-1 sample are very dense and built of largely grains without stepped structures. It confirms that the driving force of sintering just leads to the growth of randomly arranged nanoparticles and results in the formation of grains with smooth surface.^40^

Additionally, to further detail the effects of the stepped structures on cell adhesion abilities, we also tried to synthesize HA dishes with different densities of the stepped structures. Basing on the previous work,^37,38^ the particle-by-particle crystal growth mechanism is responsible for the formation of stepped structures. During the sintering process, the stepped structures are metastable and gradually disappear with the increase in sintering time. Hence, we prepared HA dishes with different densities of stepped structures by sintering the HA dishes constituted of HA mesocrystals at 1100 °C for different times. The samples are labelled as Meso-*X*, the *X* represents the hour number of sintering time. The low-magnification SEM images (Fig. S3†) show that the surfaces of all HA dishes are very dense. The corresponding high-magnification SEM images (Fig. 4b–e and S4†) show that the surfaces of all HA dishes are built of many grains, which are similar to that observed for the Meso-1 sample. However, in comparison with the Meso-1 sample, the stepped structures on the surfaces of the HA dishes decrease with the increase in sintering time, until few visible stepped structure exposes on the surface of the Meso-4 sample. On the other hand, the XRD patterns (Fig. S5†) reveal that all productions are pure HA, but their crystallization degrees are significantly increased with prolonging the sintering time.

**Fig. 5:**
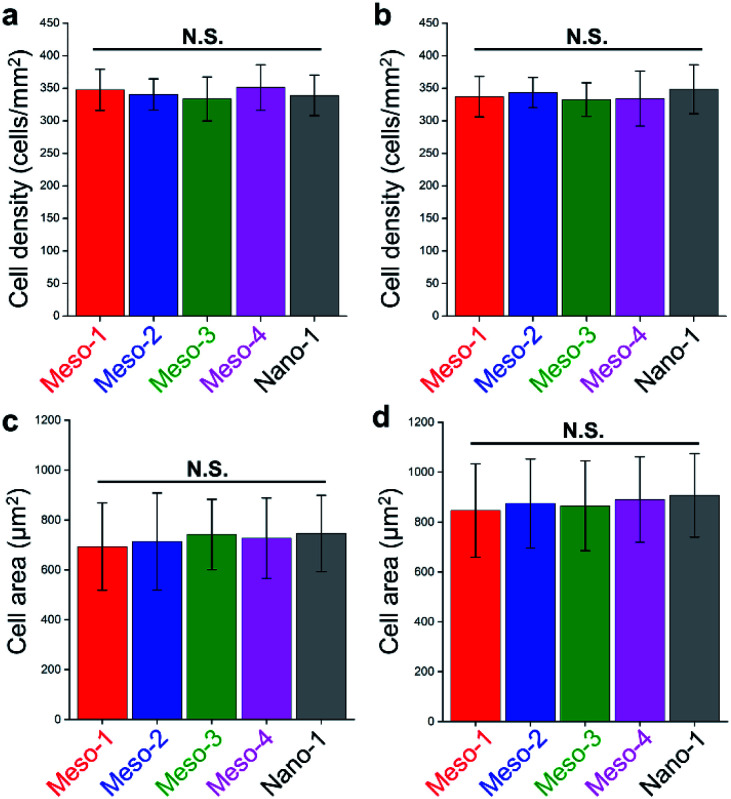
(a and b) The cell density of BMSCs cultured on different HA dish samples for (a) 1 h and (b) 3 h. (c and d) The cell spreading area of BMSCs cultured on different HA dish samples for (c) 1 h and (d) 3 h. Values were presented as mean ± s.d., N.S. represents no significant difference, compared with Nano-1 group.

The effects of the stepped structures on cell adhesion abilities were mainly evaluated by staining the F-actin cytoskeleton of BMSCs cultured on each HA dish sample for different times (1–48 h). After been cultured for 1–3 h (Fig. S6 and S7†), BMSCs are mainly round shape with small spread areas, and there is no obvious difference in the cell morphology of BMSCs on different HA dish samples. Furthermore, the number and cell spreading area of BMSCs adhering to different HA dish samples were also analyzed. As shown in Fig. 5, the cell density and cell spreading area of BMSCs attached to the surface of different HA dish samples are very close, and there is no significant statistical difference between these groups. It suggests that the stepped structures on surfaces of HA dishes does not affect the initial cell attachment.

**Fig. 6:**
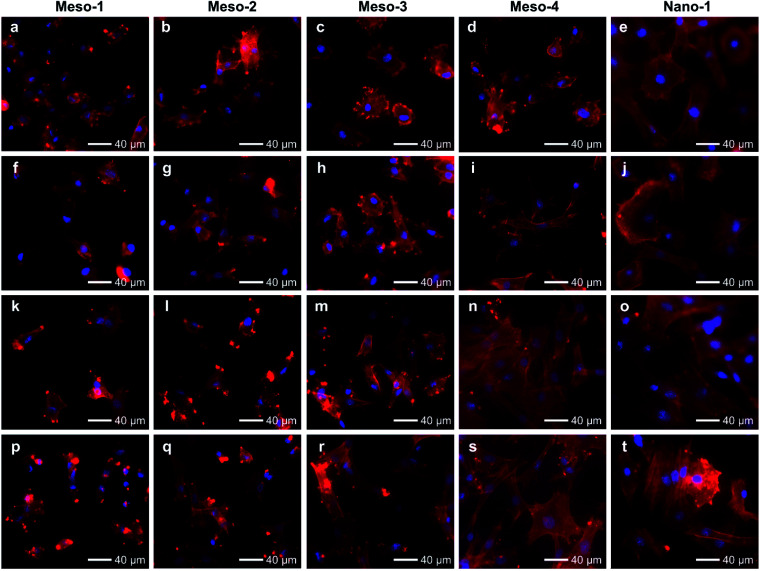
Fluorescence microscope images of BMSCs cultured on HA dish samples for different times: (a–e) 6 h; (f–j) 12 h; (k–o) 24 h; (p–t) 48 h. HA dish samples are Meso-1, Meso-2, Meso-3, Meso-4 and Nano-1 in order from left to right. The nucleus was stained blue, and F-actin was stained red.

After been cultured for 6 to 12 h (Fig. 6a–j and S8†), BMSCs adhere stably on the surface of each HA dish sample. However, the morphologies and spread areas of BMSCs are highly correlated to the density of the stepped structures on the surfaces of HA dishes. The BMSCs adhering to the Meso-1 sample show limited spread areas and have few filopodia and lamellipodia. From Meso-2 to Meso-4 and then to Nano-1 samples, the density of stepped structure decreases until the surfaces of grains are smooth; the BMSCs gradually generate more filopodia and lamellipodia, leading to well extending in polygonal shapes. It is worth noting that the cell spreading areas of BMSCs on Meso-4 and Nano-1 samples are distinctly different in 6 and 12 h time points, showing that the BMSCs cultured on Nano-1 sample seem to spread faster that on Meso-4 sample within 6 to 12 h. For this phenomenon, there may be two reasons. The first reason is that Meso-4 sample may still retain a small amount of stepped structure (Fig. 4e). The second reason is that Nano-1 sample was just sintered at 1100 °C for 1 h, while Meso-4 sample were sintered at 1100 °C for 4 h. The obvious difference in crystallization degree between Meso-4 and Nano-1 samples (Fig. S5 and S2b†) may affect the spreading rate of the cells.^41,42^ With the prolongation of culture time, the effects of the stepped structures on cell adhesion would be more obvious. After been cultured for 24 to 48 h (Fig. 6k–t and S8†), the BMSCs adhering to the Meso-1 sample are mainly spindle or round shape without obvious cell aggregation. From Meso-2 to Meso-4 and then to Nano-1 samples, the BMSCs tend to spread out fully with a larger adhesion area, and their cell morphologies gradually shift from spindle to polygonal shapes. In particular, the BMSCs adhering to the Meso-4 and Nano-1 samples show similar final morphologies, both of which perform significant cell aggregation and cell–cell junctions (Fig. 6n, o, s and t).

The above results of BMSCs adhering to different HA samples for 1–48 h suggest that BMSCs could effectively and initially attach to the surface of HA dishes in the early stage of cell attachment, regardless of whether the HA dishes expose stepped structures on surfaces or not. However, the adhesion of BMSCs cultured on the HA with stepped structures would be restricted in the process of cell spreading, and the more exposed stepped structures, the more obvious restricted adhesion.

In addition, cell adhesion is usually considered as an important index correlating with subsequent cell proliferation.^1,7,8^ Thus, the Ki67 (a nuclear marker of cell proliferation^43^) of BMSCs were stained and investigated. As shown in Fig. S9 and S10,† after being cultured for 24 to 48 h, the number of Ki67 positive cells on Nano-1 sample increase obviously, while the numbers of Ki67 positive cells on Meso-1 to Meso-4 samples just increase slightly or even do not increase. It indicates that the cell proliferations of BMSCs would be inhibited if stepped structures are exposed on the surfaces of HA dishes.

To build a convincing relationship between the stepped structure and its effects on cell adhesion of BMSCs, herein, some highly possible impacts should be excluded. Some studies have suggested that HA with low crystallinity (*e.g.*, HA that has not undergone a sintering process) would have some impacts on cell behaviours (*e.g.*, cell proliferation) due to its released ions.^1,44^ However, the HA dishes used in this work were obtained from a high temperature sintering process, resulting in high crystallinities for all samples. The Ca^2+^ ions released from the HA sample will be very small during the process of cell culture, which is confirmed by the tests of released Ca^2+^ ions (Table S1†). Thus, its effects on BMSCs are theoretically negligible. Additionally, the results of CCK-8 assay (Fig. S11†) also indicate that the BMSCs cultured in the extracts from different HA dishes show good cell viabilities and proliferations. Therefore, it is highly possible that the stepped structures on surfaces of HA dishes are responsible for the restricted adhesion of BMSCs.

For the restricted adhesion of BMSCs, we could infer some possible reasons from the effects of stepped structures on the surface chemical and physical characteristics, although the detail mechanisms of cell adhering on the surface of bioceramic materials are complex and many factors would influence the outcome of cell adhesion.^1,8,45^

Stepped structure is a kind of surface morphologies during the evolution of crystal growth and contain a large number of crystal defects (*e.g.*, lattice vacancies), which usually lead to changes in stoichiometric ratios of material surface.^15,20,25,27^ To investigate the surface elements, XPS spectra analysis were carried out. The full spectra of all HA dishes are shown in Fig. S12.† There is no evident signal from any of other impure element in addition to C atoms, and the main elements of the HA surfaces are Ca, P and O atoms. The peaks at 284.8 eV corresponds to C–C bonds, which are caused by the adventitious carbon species coming in contact with air.^46^ However, the calculated Ca/P ratio of each HA dish sample is different, and the value in order is Meso-1 (1.42) < Meso-2 (1.46) < Meso-3 (1.55) < Meso-4 (1.59) < Nano-1 (1.64), which is negatively correlated with the density of stepped structures (Fig. 4, S4 and S2d†). Given that XPS spectra analysis mainly focus on the surface characterization of materials, it suggests that the generation of stepped structures on the surfaces of HA dishes is more likely to lead to the formation of Ca^2+^ vacancies than that of PO_4_^3−^ vacancies.

**Fig. 7:**
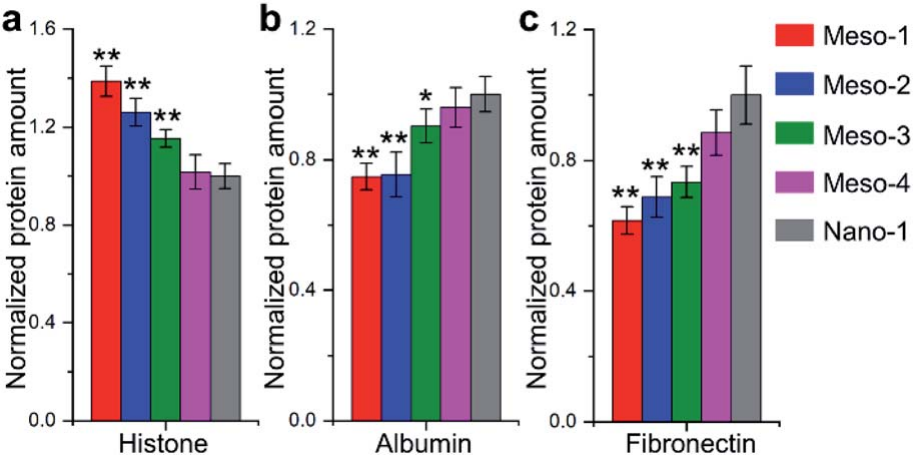
BSA assay for proteins adsorbed on the surfaces of different HA dish samples. (a) Histone; (b) albumin; (c) fibronectin. Values were presented as mean ± s.d., *n* = 5 biologically independent measurements. *P < 0.05 and **P < 0.01, compared with Nano-1.

Many theoretical studies have shown that crystal defects on HA surfaces are high reactive and can affect protein adsorption.^15,16,18,21,22^ Thus, to assess the capacities of HA dishes to adsorb proteins, BCA protein assay was applied to examine adsorptions of two model proteins with similar size and globular shape, including histone (positively charged, ∼7 nm diameter) and albumin (negatively charged, ∼8 nm diameter). As shown in Fig. 7a and b, with the decrease in density of stepped structures (from Meso-1 to Meso-4 and then to Nano-1 samples), the amount of adsorbed histone is decreased, while that of adsorbed albumin adsorption is increased. This may be due to the existence of Ca^2+^ vacancies on the stepped structures, which increases the local electronegativity of the surfaces and leads to a favourable adsorption for positively charged proteins but an unfavourable adsorption for negatively charged proteins. Among the numerous proteins that may be adsorbed on material surface, the proteins containing RGD (arginine–glycine–aspartic) sequence are thought to play a pivotal role in cell adhesion, because RGD sequence could preferentially bind to integrins of cytomembrane *via* RGD-based peptide signalling pathways.^9,47^ Hence, we examined the adsorption of fibronectin, which is one of the main components of the extracellular matrix and contains RGD sequence in its module III10.^9,47^ The results of Fig. 7c show that HA dishes would adsorb more fibronectin with the decrease in the density of stepped structures. Given that fibronectin has a negative charge at a physiological pH,^48^ the varied local electronegativity caused by Ca^2+^ vacancies on stepped structures may also be a major reason for the change of fibronectin adsorption, which is similar to the case of albumin. The negative correlation between the density of stepped structures and the fibronectin adsorption implies that the exposed stepped structure may be not conducive to fibronectin-mediated cell adhesion. The above results indicate that the generation of stepped structures could alter the Ca/P ratios of HA surface and lead to the formation of lattice vacancies. These changes in the surface chemical characteristics would affect the adsorptions of proteins, which is a possible reason for the restricted adhesion of BMSCs.

In addition, many studies have shown that some unique nano/microscale surface topographies could result in a steric barrier between cells and substrate to restrict cell adhesion.^8,45^ Stepped structures on HA surfaces comprise a large number of step edges, terraces and kinks, which form unique surface topography on the scale of ten to hundreds of nanometers (Fig. 1g and 3b–d). Thus, the exposure of stepped structures could alter the geometric topographies of HA surface and cause some changes in physical characteristics, which may be another potential reason for the restricted adhesion of BMSCs.

## Conclusions

In summary, we successfully fabricated pure HA dishes with largely stepped structures exposed on surfaces *via* an easy way starting from CaHPO_4_ and following a topotactic transformation and a sintering process. This facile method is robust and allows mass production of these HA materials, which paves the way for their large-scale practical application. Based on the principle of the method, we could easily control the densities of the stepped structures *via* altering sintering times, and also prepared HA dish sample without stepped structures. Cellular experiments reveal that the generation of stepped structures could lead to the restricted adhesion of BMSCs, and the restriction effect is highly correlated with the density of exposed stepped structures on HA surfaces. Although many underlying molecular mechanisms still require further study, this work show that controlling cell adhesion by constructing stepped structures on surfaces of HA materials is a potential strategy to regulate subsequent cell behaviours, because of the pivotal role of cell adhesion in the respond process of cells to materials. Furthermore, this strategy could theoretically cooperate with other strategies that do not need change in the surface crystal structure, such as surface micro-patterning, to further effectively and accurately regulate cell adhesion.

## Experimental

### Synthesis of HA dishes with stepped structures

All chemicals used in this work were analytical grade without further purification. This approach of synthesis HA with stepped surface was adapted from our previous work,^37^ which was divided into four steps. Firstly, two solutions were prepared by dissolving 1.134 g Ca(NO_3_)_2_·4H_2_O and 0.414 g NH_4_H_2_PO_4_ into 80 ml of 30 mM CH_3_COONa solution, respectively. After the mixture of the two solutions, 0.15 ml acetic acid was added. The reaction continued moderately stirring at 60 °C for 12 h. The obtained CaHPO_4_ particles were collected and separated by centrifugation, washed and dried. Secondly, 0.1 g CaHPO_4_ particles were added into 100 ml of 1 M NaOH solution (pre-heated to 95 °C). After reacting for 5 min, the suspensions were filtered through a 0.22 μm membrane. The resultant products were HA mesocrystal particles and were washed by consecutive centrifuging, decanting, and redispersing, in distilled water (thrice). Thirdly, 0.1 g HA mesocrystal particles were dispersed in distilled water and filtered using a vacuum-assisted filtration and nylon filtration membranes (pore size of 0.22 μm). The obtained HA samples were air-dried at room temperature for over 1 week. Fourthly, the HA dishes constituted of HA mesocrystals were heated in air up to the 1100 °C at a heating rate of 5 °C min^−1^, and held at 1100 °C for 1–4 h. The diameter of obtained HA dishes was about 10 mm.

### Synthesis of nanocrystalline HA

50 ml of 30 mM (NH_4_)_2_HPO_4_ solution was added drop by drop into 50 ml of 50 mM Ca(NO_3_)_2_·4H_2_O solution with stirring, and the pH was kept at 9.5 by adding 25 wt% NH_4_OH solution. After keeping at 80 °C for 24 h, the precipitates were collected by centrifugation, washed and dried at 60 °C.

### Characterization

Scanning electron micrographs (SEM) were obtained using a FEI Nova Nano SEM 450. Powder X-ray diffraction (XRD) studies were performed using a PANalytical X'Pert PRO diffractometer, and the average crystallite sizes were calculated according to the Scherrer equation. Transmission electron micrographs (TEM) were obtained using a FEI Tecnai G2 F30. Atomic force microscope studies were performed using a Bruker MultiMode 8. X-ray photoelectron spectra (XPS) studies were performed using a Kratos Axis Ultra DLD, and all binding energies were referenced to the C 1s peak at 284.8 eV of the surface adventitious carbon.

### Measurement of released Ca^2+^ ions

Each HA dish sample was immersed in 1 ml PBS solution (originally without Ca^2+^ ions) and incubated at 37 °C for different times (1–4 days). The Ca^2+^ concentration of each obtained extract was measured by a Ca^2+^ assay kit (Nanjing Jiancheng Bioengineering Institute), using a Multiscan Spectrum (Bio Tek, USA) and measuring the light absorbance (OD) at a wavelength of 610 nm, according to the manufacturer's instructions.

### Protein adsorption measurement

Bovine serum albumin (Sigma Aldrich), histone (Sigma Aldrich) and fibronectin (Sigma Aldrich) proteins were used and dissolved in phosphate buffered saline (PBS) solution. Then, 100 μl of protein solutions (200 μg ml^−1^) are applied on the HA dish (diameter of 10 mm) samples, respectively. After incubating at 37 °C for 2 h, the HA dish samples were carefully washed with 10 ml of PBS to remove unbound protein. BCA protein assay kit (Thermo Scientific) was used to measure the protein concentrations and the standard procedure was followed (measurements are performed five independent times, with each experiment performed in triplicate).

### Cell culture

Rat bone mesenchymal stem cells (BMSCs) were purchased from Cyagen Biosciences Inc. The BMSCs were maintained in DMEM culture medium supplemented with 10% fetal bovine serum (Giboco) and 1% penicillin/streptomycin (Giboco) at 37 °C in 5% CO_2_ prior to *in vitro* experiments.

### Evaluation of the effects of HA dish extracts on cell proliferation

Each HA dish sample was cleaned by 70% ethanol, washed by PBS twice, and placed in 24 well-plates with 1 ml DMEM culture medium. After been incubated at 37 °C for 48 h, the collected extracts were filtered through 0.22 μm Millipore syringe filter, and supplemented with 10% fetal bovine serum and 1% penicillin/streptomycin (Giboco). BMSCs suspension (100 μl) were added into each well of 48 well-plates with the concentration of 1 × 10^5^ per ml, following adding 100 μl complete culture medium. After been cultured for 12 h, the original culture medium of each well was removed, and 200 μl extracts obtained from HA dish samples were added. The cells were incubated at 37 °C for 6–144 h, and the corresponding extracts were refreshed every two days. The proliferations of BMSCs were quantified by Cell Counting Kit 8 (CCK8, Dojindo, Japan) assay, using a Multiscan Spectrum (Bio Tek, USA) and measuring the light absorbance (OD) at a wavelength of 450 nm.

### Fluorescence microscopy imaging

All of the HA dishes were cleaned by 75% ethanol, washed by PBS twice, and put in each well of 48 well-plates. Then, 200 μl BMSCs suspension were added to each well with the concentration of 2 × 10^5^ per ml, following adding 400 μl complete culture medium. After been cultured for different times (1–48 h), the culture medium of each well was removed and all samples were fixed by 4% paraformaldehyde. The samples were washed with PBS 3 times and subsequently permeabilized with 0.1% Triton X-100 (Sigma Aldrich) for 10 min, following with three washings in PBS. BMSCs were blocked with 0.5% BSA for nonspecific absorption. For cytoskeleton observations, BMSCs were stained with rhodamine-labeled phalloidin (Abcam) for intracellular actin and 4′,6-diamidino-2-phenylindole (DAPI) (Sigma Aldrich) for nuclei. For cell proliferation analyses, BMSCs were stained with Alexa Fluor 488 conjugated Ki-67 rabbit mAb (Cell Signaling Technology) to label Ki67, then DAPI were used to stain nuclei for double staining. The samples were viewed using a fluorescence microscope (Nikon, i80). The numbers and the cell spreading areas of BMSCs on the surface of HA samples were analyzed using Image J software. For cell counting, five HA dishes were measured for each group. For cell spreading area statistic, 100 cells were measured for each group.

### Statistical analyses

The data were expressed as mean ± s.d. The unpaired, two-sided Student's *t*-test was applied for variance analysis. *P < 0.05 was considered significant, **P < 0.01 was considered highly significant, and N.S. was considered as no significant difference.

## Author contributions

The manuscript was written through contributions of all authors. All authors have given approval to the final version of the manuscript.

## Conflicts of interest

There are no conflicts to declare.

## Supplementary Material

RA-012-D2RA00756H-s001
