# Supplementary material for: The restricted adhesion of bone marrow mesenchymal stem cells by stepped structures on surfaces of hydroxyapatite
Source: RSC Adv. 2022 Apr 20;12(19):12002–10. doi: 10.1039/d2ra00756h (PMC9019829; doi:10.1039/d2ra00756h)
Supplement: RA-012-D2RA00756H-s001 [file RA-012-D2RA00756H-s001.pdf]

Electronic Supplementary Material (ESI) for RSC Advances.
This journal is © The Royal Society of Chemistry 2022

Supplementary Information

The restricted adhesion of bone marrow mesenchymal stem cells by stepped structures on surfaces of hydroxyapatite

Jin Chen,[#a,b] Zhuo Huang,[#a] Fang Wang,[b] Min Gong,[a] Xueli Zhang,[b] Yajing Wang,[c] Zuquan Hu,[*a] Zhu Zeng[*a,b] and Yun Wang[*a,b]

[a] Key Laboratory of Biology and Medical Engineering/Immune Cells and Antibody Engineering Research Center of Guizhou Province, School of Biology and Engineering, Guizhou Medical University, Guiyang, 550025, P. R. China.

[b] Key Laboratory of Infectious Immune and Antibody Engineering of Guizhou Province, School of Basic Medical Sciences, Guizhou Medical University, Guiyang, 550025, P. R. China.

[c] The Affiliated Stomatological Hospital of Guizhou Medical University, Guizhou Medical University, Guiyang, 550025, P. R. China.

[#] These authors contributed equally to this work.

AUTHOR INFORMATION

Corresponding Author

* huzuquan@gmc.edu.cn;

* zengzhu@gmc.edu.cn;

* wangyun@gmc.edu.cn；

Results

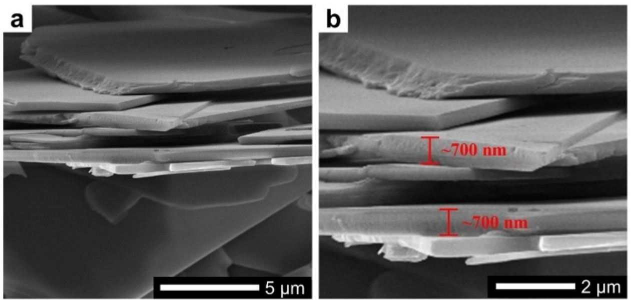

Fig. S1 SEM images of $CaHPO_4$ particles in cross-section view. (a) Low magnification. (b) High magnification.

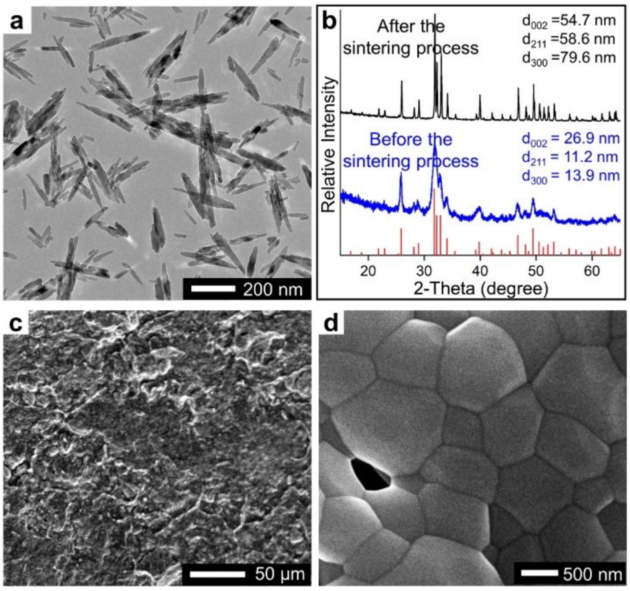

Fig. S2 (a) TEM image of HA nanoparticles. (b) XRD patterns of the HA nanoparticles and the Nano-1 dishes. (c) and (d) SEM images of Nano-1 dishes.

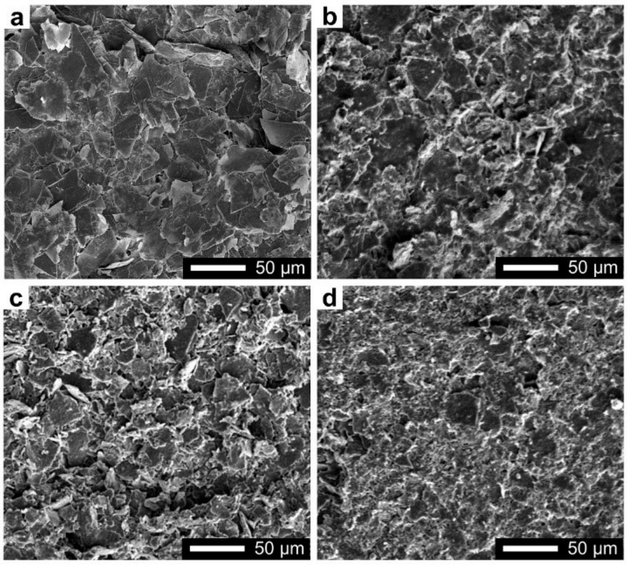

Fig. S3 Low magnification SEM images of different HA dish samples: (a) Meso-1; (b) Meso-2; (c) Meso-3; (d) Meso-4.

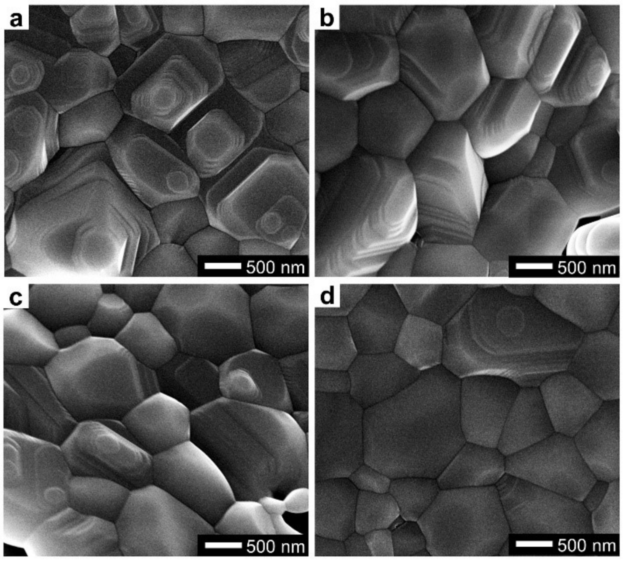

Fig. S4 High magnification SEM images of different HA dish samples: (a) Meso-1; (b) Meso-2; (c) Meso-3; (d) Meso-4.

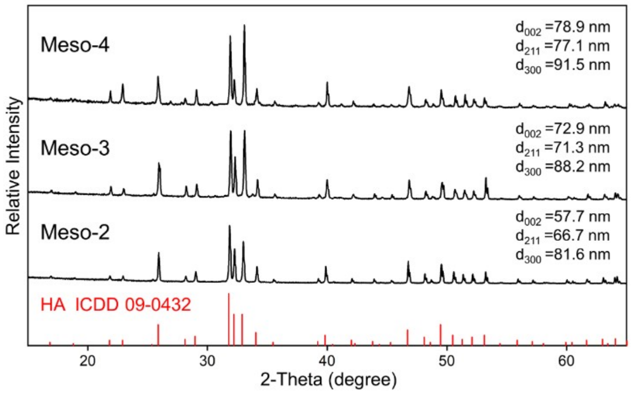

Fig. S5 XRD patterns of different HA dish samples.

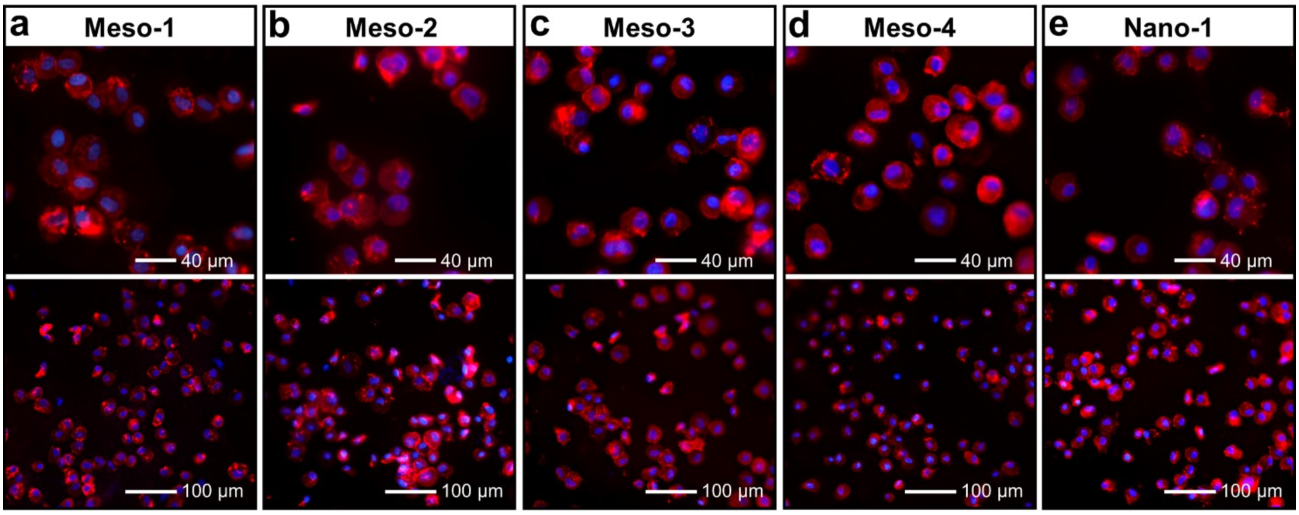

Fig. S6 Fluorescence microscope images of BMSCs cultured on different HA dish samples for 1 h: (a) Meso-1, (b) Meso-2, (c) Meso-3, (d) Meso-4, (e) Nano-1; the nucleus was stained blue, and F-actin was stained red.

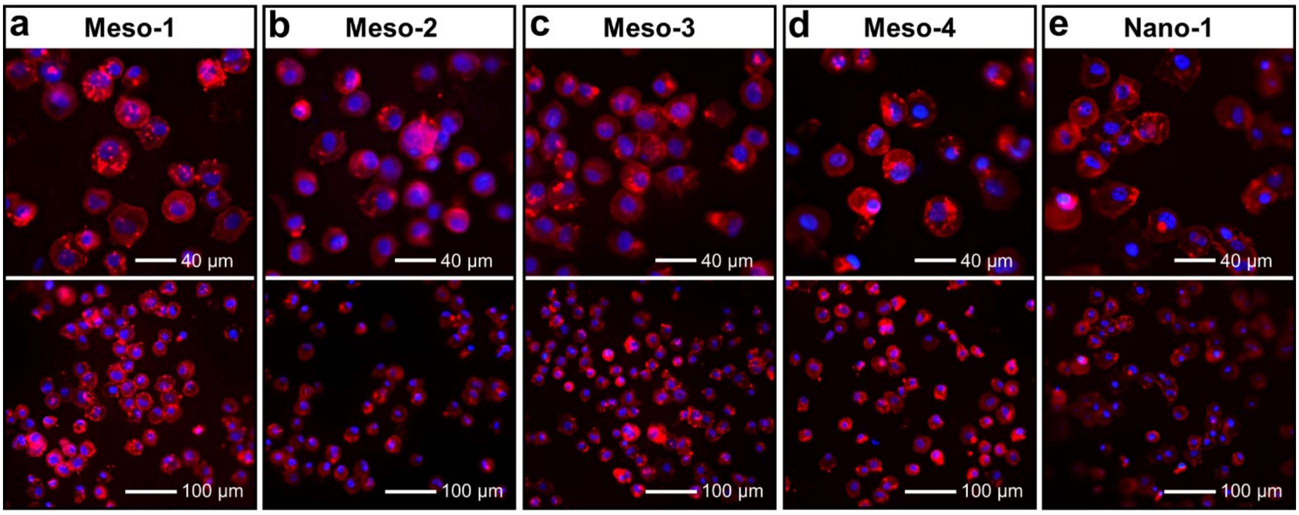

Fig. S7 Fluorescence microscope images of BMSCs cultured on different HA dish samples for 3 h: (a) Meso-1, (b) Meso-2, (c) Meso-3, (d) Meso-4, (e) Nano-1; the nucleus was stained blue, and F-actin was stained red.

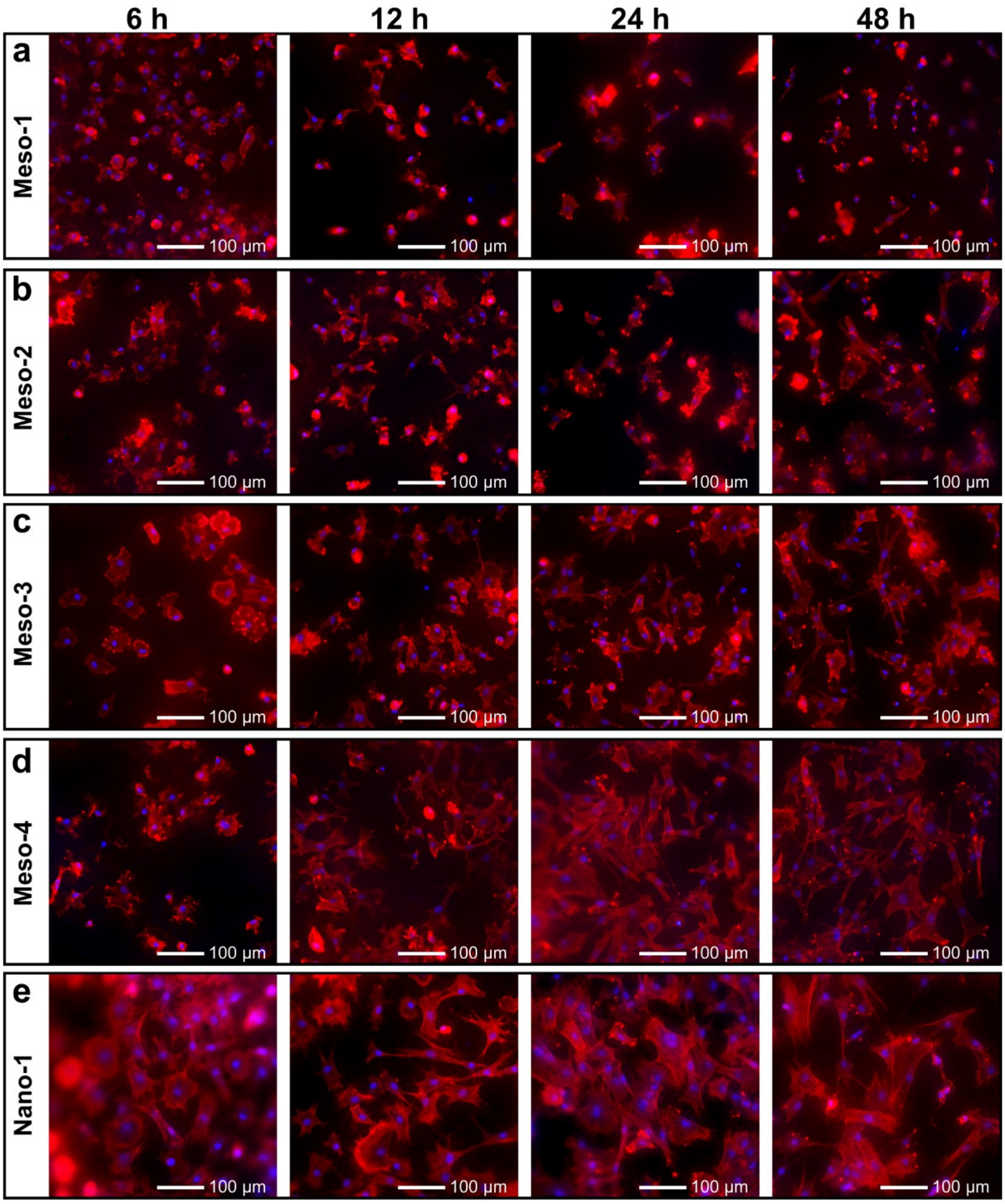

Fig. S8 Low magnification fluorescence microscope images of BMSCs cultured on different HA dish samples for 6, 12, 24 and 48 h: (a) Meso-1, (b) Meso-2, (c) Meso-3, (d) Meso-4, (e) Nano-1; the nucleus was stained blue, and F-actin was stained red.

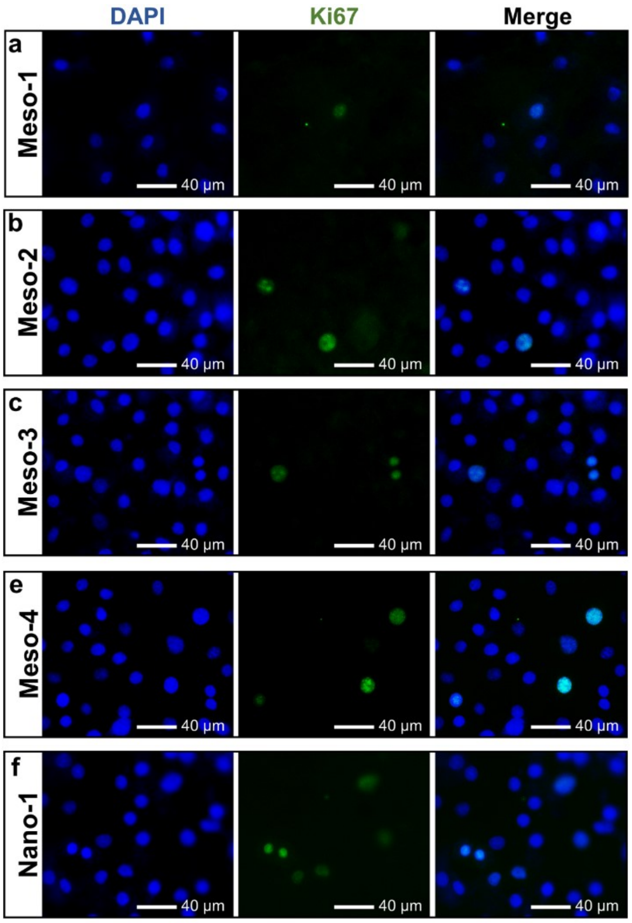

Fig. S9 Fluorescence microscope images of BMSCs cultured on different HA dish samples for 24 h: (a) Meso-1, (b) Meso-2, (c) Meso-3, (d) Meso-4, (e) Nano-1; the nucleus was stained blue, and Ki76 was stained green.

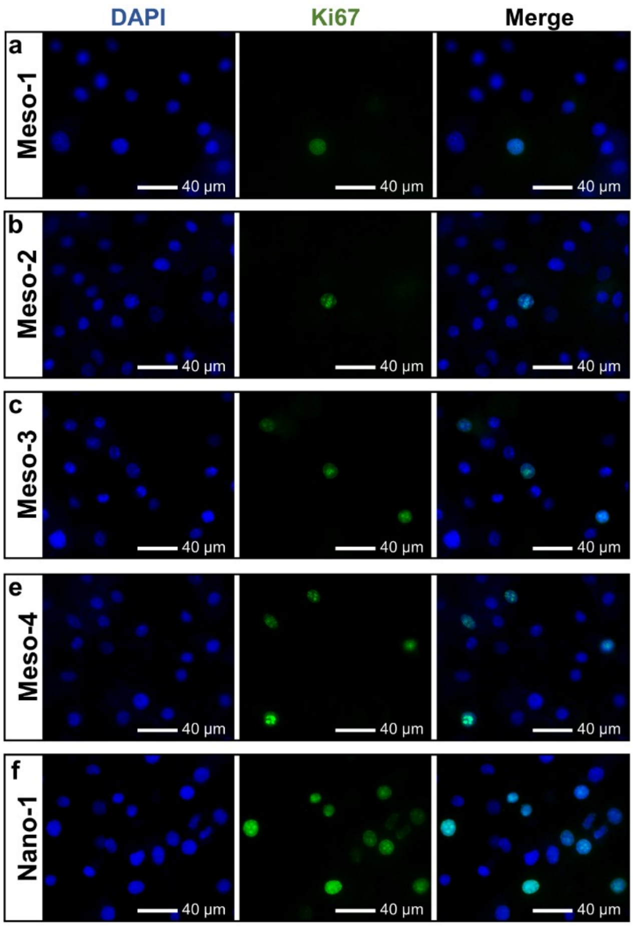

Fig. S10 Fluorescence microscope images of BMSCs cultured on different HA dish samples for 48 h: (a) Meso-1, (b) Meso-2, (c) Meso-3, (d) Meso-4, (e) Nano-1; the nucleus was stained blue, and Ki76 was stained green.

Samples	1 day	2 days	4 days
Meso-1	< 0.01 mM	< 0.01 mM	< 0.01 mM
Meso-2	< 0.01 mM	< 0.01 mM	< 0.01 mM
Meso-3	< 0.01 mM	< 0.01 mM	< 0.01 mM
Meso-4	< 0.01 mM	< 0.01 mM	< 0.01 mM
Meso-5	< 0.01 mM	< 0.01 mM	< 0.01 mM

Table S1 Concentrations of Ca^{2+} ions in PBS solution samples obtained by incubating different HA dish samples in 1 ml PBS solution (originally without Ca^{2+} ions) at 37 °C for different times (1~4 days).

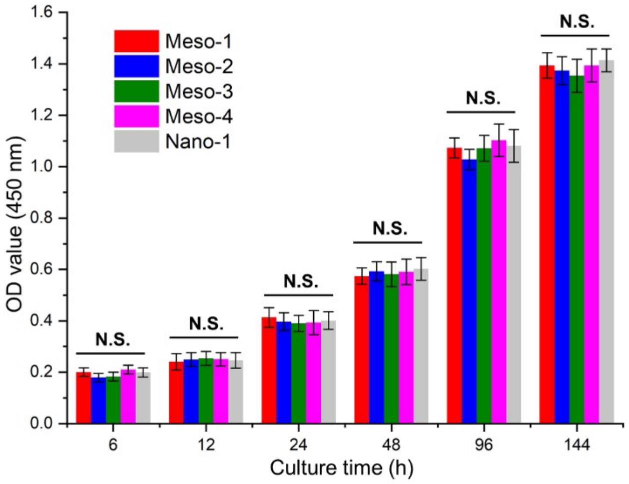

Fig. S11 CCK-8 assay for the proliferation of BMSCs treated by the extracts from different HA dish samples. Values were presented as mean ± s.d., n = 5 biologically independent measurements, N.S. represents no significant difference, compared with Nano-1 group.

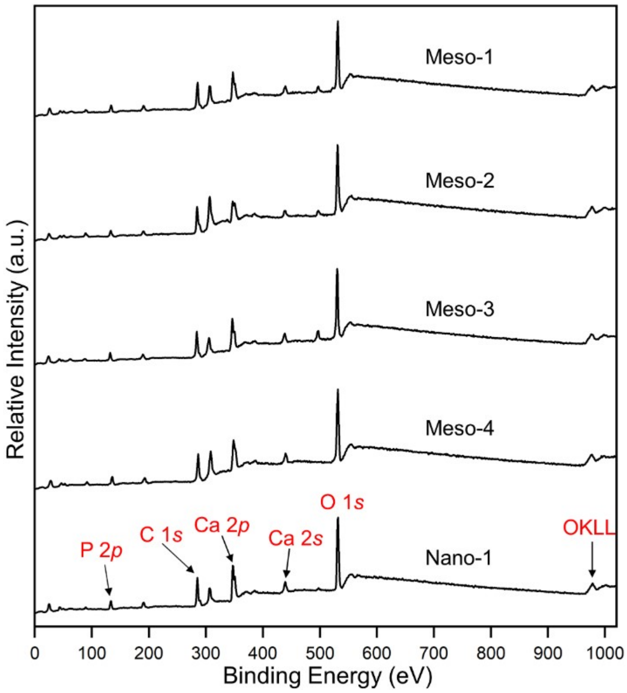

Fig. S12 XPS full spectra of different HA dish samples.
